# Comparative Study of Injected Alzheimer’s Disease Models in Rats: Insights from Experimental Research

**DOI:** 10.3390/pathophysiology31040047

**Published:** 2024-11-20

**Authors:** Hanane Doumar, Hicham El Mostafi, Aboubaker Elhessni, Abderrahim Laaziz, Abdelhalem Mesfioui

**Affiliations:** Biology and Health Laboratory (BHL), Department of Biology, Faculty of Sciences, Ibn Tofail University, Kenitra 14 000, Morocco; doumarhanan89@gmail.com (H.D.); elhessni70@yahoo.fr (A.E.); ab.laaziz1@gmail.com (A.L.); a.mesfioui@yahoo.fr (A.M.)

**Keywords:** Alzheimer’s disease, cognitive decline, neurodegenerescence, animal model, rat

## Abstract

Background/Objectives: Alzheimer’s disease (AD) remains incurable, highlighting the need for new and diverse animal models to better understand its complex mechanisms. This study compares various injected animal models of AD, focusing on the main theories that explain the disease; Methods: Female Wistar rats (10-months old) were administered intracebroventricularly by artificial cerebrospinal fluid (aCSF) (Control), beta amyloid Aβ1-42 (BA), okadaic acid (OKA), lipopolysaccharides (LPS), buthionine sulfoximine (BSO) or by a mixture of these different molecules (MLG). Cognitive performance was assessed one week or one month after stereotaxic surgery; Results: Our results, show that only the Aβ and the MLG induced a persistence and progressive deficits in the working memory, recognition memory and spatial memory in rats. As the hippocampus (HIP) and the prefrontal cortex (PFC) are particularly involved in memory behavior, we analyzed long-term neuroadaptations in these brain subregions using spectrophotometric and histological methods to assess oxidative stress changes and neuronal loss, respectively. We found that the behavioral impairments in memory and learning were accompanied by irreversible oxidative stress changes and neurodegenerescence, particularly in the HIP; Conclusions: This study provides promising data on the modeling of AD in order to develop an effective therapeutic approach.

## 1. Introduction

Alzheimer’s disease (AD) is a progressive neurodegenerative disorder resulting in debilitating cognitive decline that affects approximately 46 million people worldwide [1]. Despite extensive research, the precise mechanisms underlying AD remain elusive. The diversity and uncertainty of the pathogenesis of AD have caused difficulties in the development of effective treatment, and most of the clinical and preclinical trials performed in recent decades have failed. Developing an animal model that recapitulates various aspects of AD, including progressive and irreversible deficits in cognitive function, represents a valuable tool for facilitating efficient in vivo screening. These animal models should be designed based on the various pathophysiological hypotheses of the disease, including beta-amyloid (Aβ) plaque aggregation, tau protein hyperphosphorylation, oxidative stress, and an exacerbated neuro-inflammatory response [2].

Neurofibrillary tangles (NFTs) and amyloid plaques, particularly those formed by Aβ1-42 aggregates, are considered the two primary pathological features in AD, especially in its inherited forms. According to the amyloid hypothesis, the peptide Aβ1-42 is produced from the amyloid precursor protein (APP) through the activity of β- and γ-secretases. Under normal conditions, APP is cleaved by α- or β-secretases, leading to non-toxic fragments. However, when APP is cleaved sequentially by β-secretase followed by γ-secretase, the result is the neurotoxic Aβ1-42 peptide. Elevated levels of Aβ1-42 contribute to the formation of amyloid plaques, which in turn promote neurotoxicity. This peptide encourages the aggregation of fibrillar amyloid deposits, known as neuritic plaques, rather than the normal breakdown of APP [3]. The accumulation of these plaques triggers microglial and astrocytic activation, oxidative damage, tau aggregation, and ultimately neuronal loss, leading to the synaptic dysfunction characteristic of AD [4]. These pathological mechanisms have been extensively investigated in rodent models, particularly in those induced by stereotaxic intracerebroventricular (icv) injection of Aβ1-42 oligomers [5].

The hyperphosphorylation of tau protein is another hallmark of AD. Tau, a microtubule-associated protein, becomes abnormally phosphorylated in AD, leading to impaired microtubule stability and neuronal dysfunction [6]. The disruption of the normal balance between tau phosphorylation and dephosphorylation is critical to AD [7]. Okadaic acid (OKA), a potent and selective inhibitor of protein phosphatases PP2A and PP1, induces tau hyperphosphorylation and accumulation, mimicking AD-like tau pathology in both in vitro and in vivo models [8,9]. In addition to driving tau hyperphosphorylation, OKA also promotes oxidative stress in various brain regions, exacerbating the neurodegenerative process. Experimental studies in rats have shown that OKA administration results in memory deficits, further supporting the link between tau pathology and cognitive impairment in AD [8].

In addition to tau aggregation and Aβ1-42 accumulation, oxidative stress is a key contributor to the progression of AD. The excessive production of reactive oxygen species (ROS) leads to oxidative damage in neurons, contributing to synaptic dysfunction and neuronal loss [10]. The imbalance between ROS production and the brain’s ability to neutralize them through antioxidant enzymes, such as superoxide dismutase (SOD) and catalase (CAT), plays a significant role in AD pathophysiology. Numerous studies have demonstrated oxidative damage in the brains of AD patients, including increased lipid peroxidation products, protein oxidation, and oxidative damage to nucleic acids [11,12,13,14,15]. Studies have also shown that the use of oxidative stress inducers, such as Fe^2+^ and buthionine sulfoximine (BSO), in animal models leads to neuronal cell death, amyloid deposition, gliosis, and memory impairment, further highlighting the role of oxidative stress in AD progression [16].

Neuroinflammation is another key mechanism involved in the development of AD, acting as a brain immune defense system [17,18]. This immune response triggers the infiltration of glial cells by circulating immune cells and the release of pro-inflammatory molecules, such as tumor necrosis factor (TNF)-α, interleukins (IL)-1β and IL-6, nitric oxide, prostaglandin E2 (PGE2), chemokines, and ROS. These glial-derived cytokines can bind to specific receptors on the surface of neurons, activating apoptotic signaling pathways. For instance, TNF-α has been shown to bind to the TNF receptor 1 (TNFR1), initiating neuronal apoptosis [19]. Among the various animal models used to study neuroinflammation in AD, the lipopolysaccharide (LPS)-induced model is one of the most commonly employed [20].

To investigate the individual and combined effects of these pathological mechanisms in AD, we developed a comparative injected model of AD in rats. This study examines the roles of Aβ plaque aggregation, tau protein phosphorylation, oxidative stress, and neuroinflammation by administering specific molecules known to induce these disorders in the brain: Aβ1-42, OKA, LPS, and BSO, respectively. We assess their impacts on (i) working memory, recognition memory, and spatial performance memory; (ii) oxidative status in the hippocampus (HIP) and prefrontal cortex (PFC) brain subregions; and (iii) neurodegeneration in the prelimbic cortex (PrL), medial cortex (M1), hippocampal pyramidal cell layer (CA3) and dental gyrus (DG). Additionally, we investigate for the first time the synergistic effects of these four molecules when combined, aiming to induce AD-like cognitive deficits, particularly targeting the HIP and PFC. Through this model, we aim to provide a comprehensive understanding of how these pathological processes interact and synergize to impact cognitive functions both in the short and long term in Wistar rats, thereby advancing the development of targeted therapeutic strategies for AD.

## 2. Materials and Methods

### 2.1. Animals and Ethics Statement

One hundred twenty Female Wistar rats born in our animal facility (Ibn Tofail University, Kénitra, Morocco) were bred and housed collectively in a temperature- and humidity-controlled room (21 ± 1 °C and 55–60%, respectively) under a 12 h light/dark cycle (lights on at 7:00). They had free access to food and water. Animals were 10 months old at the beginning of the experiments and were housed 5 per cage. All experimental procedures were performed according to the NIH Guide for the Care and Use of Laboratory Animals and approved by the Institutional Review Board of the Center for Doctoral Studies of the Faculty of Sciences, Ibn Tofail University (Approval date: 1 September 2024). Efforts were made to minimize animal suffering and to reduce the number of the animals used.

**Figure 1:**
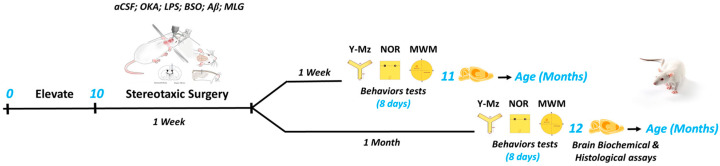
Experimental timeline.

The choice of female rats and the age of 10 months were made to ensure the best model for studying AD, given that clinical studies show a higher prevalence and progression rate of AD in women [21]. Additionally, 10 months is a suitable age for modeling the sporadic form of AD, as it aligns with the increased risk factors observed in human populations over 60 years of age [22]. The rats were assigned randomly to six groups, and were administered intracebroventricularly in the Standard stereotaxic apparatus (RWD Life Science, Shenzhen, China) by artificial cerebrospinal fluid (aCSF) (control group: CTR), Aβ oligomeric Aβ1-42 (BA group), okadaic acid (OKA group), lipopolysaccharides (LPS group), buthionine sulfoximine (BSO group) or by a mixture of these different molecules (MLG group). Animals were divided into two cohorts (60 each, with 10 rats per group). Cognitive performance was assessed one week (Cohort 1) or one month (Cohort 2) after stereotaxic surgery. For both cohorts, the animals in each group were further subdivided into two (5 each) for histological evaluation or oxidative stress assessment in the brain. Training and testing were done during the light phase between 9:00 and 15:00. The experimental procedures are summarized in Figure 1.

Female rats, aged 10 months at the start of stereotaxic surgery, were administered intracebroventricularly with artificial cerebrospinal fluid (aCSF) (10 µL), beta-amyloid Aβ1-42 (BA), okadaic acid (OKA), lipopolysaccharides (LPS), or buthionine sulfoximine (BSO), as well as a mixture of these substances at equal volumes (MLG). Following a recovery period of one week from surgery and the effects of anesthetic drugs, the animals were randomly divided into two cohorts of 60 each, with 10 rats per group. Cognitive performance was evaluated using the Y-Maze (Y-Mz), Novel Object Recognition (NOR), and Morris water maze (MWM) tests, one week (Cohort 1) or one month (Cohort 2) after the surgery. Immediately following the behavioral assessment period (8 days), histological (n = 5) and oxidative stress (n = 5) evaluations were conducted on the rats’ brains for both cohorts.

### 2.2. Stereotaxic Surgery

At ten months of age, rats underwent stereotaxic, sterile-tip surgery. Rats were anesthetized with 7% chloral hydrate (0.5 mL/kg, intraperitoneally). The rat’s cranial skull was secured in a prone position on a stereotaxic apparatus using ear bars and a mouthpiece positioned behind the incisors to immobilize the head, ensuring no movement of the skull. An ophthalmic ointment was applied to the animal’s eyes to prevent dryness and infection during surgery. Before making the skin incision, the cranial skin was disinfected with cotton soaked in a disinfection solution. A 1 cm incision was made in the bregma region using a razor blade.

The coordinates of the lateral ventricle (LV) were determined using the Paxinos-Watson stereotaxic atlas (2013) [23] with the following coordinates: Anteroposterior (AP): −1.3 mm. Mediolateral (ML): +1.7 mm. The skull was carefully drilled at the injection site to avoid damaging the dura mater, which was then incised with a sterile needle. The Hamilton syringe (10 µL), filled with the prepared solution, was lowered to the skull surface. The depth coordinates (dorsoventral) were calculated using the atlas: Dorsoventral (DV): −3.5 mm. 10 µL of the prepared solution was injected at a rate of 1 µL/min.

### 2.3. Preparation of Molecules

All chemicals used in this study were purchased from HexaBiogen (Marrakesh, Morocco). The oligomer Aβ1-42 rat peptide (orb216484-1 mg), BSO (SC200824-500 mg), LPS (SC286136-5 mg) and OKA (SC202260-25 µg) were dissolved in aCSF (147 mmol/L NaCl, 2.9 mmol/L KCl, 1.6 mmol/L MgCl_2_, 1.7 mmol/L CaCl_2_, and 2.2 mmol/L dextrose pH 7.4)).

Aβ1-42. The oligomeric form of Aβ1-42 was prepared as follows: A 1 mg lyophilized powder was equilibrated for 30 min at room temperature before opening. To dissolve the powder, 75 µL of 1% NH_4_OH was added, followed by 925 µL of aCSF to achieve a final concentration of 1 mg/mL. The mixture was vortexed, aliquoted into Eppendorf tubes, and stored at −80 °C until use. The objective was to obtain Aβ1-42 assemblies with an optimal size of 8 to 10 nm in height, as referenced in a previous study [24]. To achieve this, a 200 µM solution of Aβ was aggregated over 24 h. Samples were sonicated in a bath sonicator for 5 min and subsequently diluted to a final concentration of 10 µM before icv injection (see Stereotaxic Surgery).

BSO. A solution containing 12 mM BSO and 1 mM ferrous sulfate (FeSO_4_) was prepared with a pH of 5.1, as referenced in [16]. These solutions were diluted in aCSF prior to use.

OKA. A solution of OKA was prepared at a concentration of 20 ng/µL. A volume of 10 µL, corresponding to a dose of 200 ng per rat, was injected icv as per the protocol described in [25].

LPS (*Escherichia coli*, L3129). A solution of LPS was prepared at a concentration of 1 µg/µL, as referenced in [26].

MLG. The mixture solution, which included Aβ1-42 oligomers, BSO with FeSO_4_, OKA, and LPS, was prepared by dissolving each component in equal volumes from their respective stock solutions. The prepared solution was freshly prepared before the icv injections.

### 2.4. Behavioral Assays

One week or one month after stereotaxic surgery, the rats underwent selected behavioral tests to evaluate cognitive impairment. In this study, all animals were exposed to three tests over eight days, in the following order: Y-maze, object recognition, and water maze tests. For habituation, the animals were placed in the testing equipment for one hour before each test. All behaviors were recorded with a camera for subsequent analysis.

Y-Maze spontaneous alternation (Y-Mz) test. The Y-Mz test is widely used to assess impairments in working memory in animals. The Y-Mz apparatus consists of three arms, each measuring 50 cm in length, 10 cm in width, and 18 cm in height. The arms are connected at central open ends with a 120° angle between each pair of arms. In the procedure, a rat is placed at the center of the maze and is allowed to explore freely for a period of 5 min. During this time, the number of entries into each arm and the sequence of arm entries are recorded every 5 min using a video camera. A successful spontaneous alternation is defined as three consecutive entries into different arms. The alternation rate is calculated as follows: percentage of spontaneous alternations (%SPA) = (number of successful alternations/(N − 2)) × 100%. The expected chance level is approximately 22% SPA.

Novel objects recognition (NOR) test. The NOR test is used to assess mid-term recognition memory. The test is performed in an open square arena measuring 50 cm × 50 cm × 60 cm. It includes three phases: adaptation, training, and testing. During the training phase, two identical objects are placed along the centerline of the arena, and the rat is allowed to explore for 5 min. After a 24-h interval, one of the objects is replaced with a novel object, and the rat is given another 5-min exploration period. The time spent exploring the familiar object (TF) and the novel object (TN) is recorded. The memory of the rat is evaluated by calculating the Recognition Index (RI), defined as RI = TN/(TN + TF). A 50% recognition index indicates random exploration, and a higher index reflects better memory performance.

Morris water maze (MWM) test. The MWM is used to evaluate spatial learning and memory. In this test, rats are trained to locate a hidden platform submerged in opaque water by using external visual cues [27]. The maze consists of a circular tank with a diameter of 1.50 m, filled with water maintained at a temperature of 21 ± 1 °C. The platform, 20 cm in diameter, is retractable and is hidden below the surface. The rats undergo 5 consecutive days of training with 5 trial blocks each day. Each rat starts from one of four randomized positions (North, South, East, or West) and must locate the hidden platform. Each trial lasts up to 120 s, with intervals of 15–20 min between trials, and the latency to reach the platform is recorded. Spatial learning performance is assessed through a probe trial conducted one hour after the final training session (on day 5), during which the target platform is removed. The pool is divided into four equal quadrants: the target quadrant, the adjacent right and left quadrants, and the opposite quadrant. Performance is evaluated based on the time spent in the target quadrant and the number of times the rat crosses the location where the platform was previously located. Increased time spent in the target quadrant and crossing the platform’s theoretical location indicate better memory retention.

### 2.5. Brain Tissue Histology Evaluation

After behavioral testing, animals are deeply anesthetized with 7% hydrate choral (5 mL/kg, i.p.) and transcardially perfused with heparinized saline (0.9% NaCl with 1000 IU heparin) for 2 min, followed by 4% paraformaldehyde in 0.1 M phosphate-buffered saline (PBS, pH 7.4). The brains are extracted and post-fixed for 12 h in the same fixative, then cryoprotected in 20% sucrose/0.1 M PBS at 4 °C for 24 h. The following day, 30-μm serial sections are made using a vibratome (VT1000 S, LEIKA Biosystems, Nussloch, CA, USA) and stored in a glycerol solution at 4 °C. The sections are mounted on gelatin-coated slides and dried overnight. Staining is carried out with Cresyl violet for histological analysis as described previously [28]. Neurons in the prefrontal cortex (PrL), motor cortex (M1), CA3 hippocampal region, and dentate gyrus (DG) are observed under an optical microscope using an HDMI camera (B-500TiFL, OPTIKA S.r.l., Pomerania, Italy). Morphological differences are evaluated at magnifications of 10×, 20×, and 40×.

### 2.6. Brain Neurodegeneration Evaluation

Neurodegeneration was assessed by examining Nissl-stained coronal brain sections. The severity of neuronal damage was scored using a semi-quantitative grading system previously outlined [29]:

- Score 0: no observable damage,
- Score 1: minor structural abnormalities without obvious neuronal loss,
- Score 2: lesions affecting 20–50% of neurons,
- Score 3: lesions involving more than 50% of neurons.

This grading system was applied to the PrL, M1, CA3, and DG regions (Figure 4A), with four sections per rat being scored [23]. The average scores from both hemispheres were used to calculate group data. Additionally, the number of polymorph neurons in these regions was quantified using ImageJ 1.45 software (Microsoft Java, Redmond, WA, USA) [30]. Neurons with a diameter greater than 8 μm and characteristic neuronal morphology were counted. For each rat, three sections were analyzed, and the neuron counts were averaged across the sections for each brain region.

### 2.7. Brain Oxidative Stress Evaluation

To assess oxidative stress, rats were sacrificed by decapitation at the end of the behavioral testing period. The brains were rapidly removed and cooled on dry ice. The hippocampus (HIP) and prefrontal cortex (PFC) were dissected on ice using a rat brain atlas (Figure 3A). Brain tissues were homogenized in ice-cold 20 mM Tris-HCl buffer (pH 7.4) for subsequent analyses of lipid peroxidation (LPO), nitrite levels (NO), and activities of catalase (CAT) and superoxide dismutase (SOD). Oxidative stress parameters in the HIP and PFC were quantified using spectrophotometric methods as previously described [31].

- NO assay: Performed using the Griess reagent (0.1% *N*-(1-naphthyl) ethylene diamine dihydrochloride; 1% sulfanilamide in 5% phosphoric acid; 1:1 ratio), with results expressed as μmol of nitrite per gram of homogenate.
- LPO assay: Lipid peroxidation was assessed by measuring thiobarbituric acid-reacting substances, with results reported as nmol of malondialdehyde (MDA) per gram of wet tissue.
- CAT activity: Measured by the decomposition of hydrogen peroxide (H_2_O_2_), with results expressed as mmol of H_2_O/min/mg of protein.
- SOD activity: Evaluated by its ability to inhibit the photoreduction of nitroblue tetrazolium, with results expressed as mmol/min/mg of protein.

### 2.8. Statistical Analysis

Statistical analyses were performed using GraphPad Prism 8.01 (GraphPad Software Inc., La Jolla, CA, USA). Data are presented as mean ± standard error of the mean (SEM) and were analyzed using one-way or two-way ANOVA. When significant differences were detected, post hoc Tukey tests were used to determine the specific group differences. For non-parametric data from semi-quantitative histological analyses, Kruskal-Wallis ANOVA was applied, followed by Dunn’s post hoc test. Statistical significance was set at *p* < 0.05. To assess variations in the parameters across the different tests, a Z-score was calculated using the following formula:

$$Z=\frac{X-\mu }{\sigma }$$

where: Z is the Z-score, *X* is the value of the parameter being evaluated, *μ* is the mean of the parameter values in the control group, *σ* is the standard deviation of the parameter values in the control group.

This standardization allows for the comparison of data across different tests by converting them into a common scale. The Z-score indicates how many standard deviations a data point is from the mean of the control group. A positive Z-score signifies that the data point is above the mean, while a negative Z-score indicates that it is below the mean.

The Z-score indeed quantifies variations in the studied parameters relative to the mean and standard deviation of a reference group. A factor of −1 is applied when the direction of variation of the parameter is opposite to the magnitude of the symptom it measures. These Z-scores were subsequently aggregated into categories of disorders according to the method used by Guilloux et al. (2011) [32]. In our study, to calculate the Z-score for cognition, different memory disorders were aggregated using the following formula:

$$Z-score\;Cognition=\frac{Z\%SPA+Z\%IR+Z\%TQC}{Number\;of\;tests}$$

where: *SPA*: spontaneous alternation in the Y-Mz test; *IR*: Recognition Index in the NOR test; *TQC*: Time in quadrant Cible in the MWM test.

A Z-score was also calculated for each experimental group, considering it as an indicator of the corresponding pathophysiological hypothesis for AD. For example, the Z-score for the Aβ accumulation hypothesis corresponds to the average of the Z-scores for all behavioral parameters (Cognition Z-score), biochemical parameters (Oxidative Stress Z-score), and histological parameters (Neurodegeneration Z-score), according to the following formula:

$$Z-score\;A\beta \;Oligomeric\;Hypothesis=\frac{Z\;Cognition+Z\;Oxidative\;stress+Z\;Neurodegeneration}{Number\;of\;parameter\;categories}$$

## 3. Results

### 3.1. Body Weight Gain

No significant differences in weight gain between treatment groups were noted during the study (group: F(4.190) = 2.54, *p* = 0.112). All animals exhibited an increase in body weight during the treatment period (day: F(1.190) = 179, *p* < 0.001). The mean body weight gains for the period was 215 ± 1.8 g in CTR group, 230 ± 2.6 g in OKA group, 216 ± 2.1 g in LPS group, 194 ± 3.3 g in BSO group, 229 ± 0.5 in BA group and 188 ± 4.6 g in MLG group.

### 3.2. Behavioral Assays

To evaluate cognitive deficits in the rat models, we used the Y-Maze, the Novel object recognition and the Morris water maze tests.

#### 3.2.1. Effects on Working Memory in the Y-Mz Test

Following the first week of the recovery period, the Y-Maze test indicated a notable decrease in SPA for rats in the BA and MLG groups compared to the CTR group (*p* = 0.035 and *p* = 0.007, respectively). However, after one month, SPA showed improvement in the BA group and a more pronounced decrease in the MLG group compared to the CTR group (*p* = 0.310 and *p* = 0.0002, respectively).

**Figure 2:**
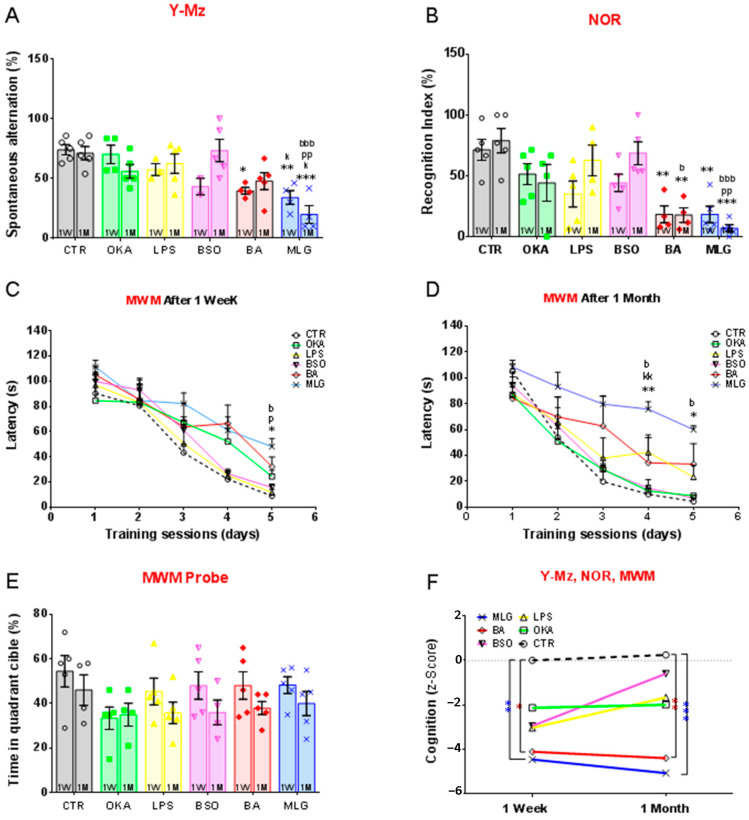
Cognitive performance in the Y-Maze (Y-Mz), Novel Object Recognition (NOR) and Morris water maze (MWM) tests. These tests were performed 1 week (1W) or 1 month (1M) after the stereotaxic surgery in rats injected by aCSF (CTR), beta amyloid Aβ1-42 (BA), okadaic acid (OKA), lipopolysaccharides (LPS), buthionine sulfoximine (BSO) or by a mixture of these different molecules (MLG). Bars represent the mean ± SEM. of (**A**) the Y-maze spontaneous alternation (%), (**B**) the recognition index (%), (**C**,**D**) the latency to reach the hidden platform (s) in the MWM, one week and one month after stereotaxic surgery, respectively, (**E**) the time in the quadrant cible assessed during a probe trial of the MWM, (**F**) The Cognition Z-score was calculated based on the animals’ performances in the three preceding behavioral tests. Symbols Signification: * *p* < 0.05, ** *p* < 0.01, *** *p* < 0.001 vs. CTR; ^k^ *p* < 0.05, ^kk^ *p* < 0.01 vs. AOK; ^p^ *p* < 0.05, ^pp^ *p* < 0.01 vs. LPS; ^b^ *p* < 0.05, ^bbb^ *p* < 0.001 vs. BTN according to two-way ANOVA followed by Tukey’s test.

In comparison to the OKA, LPS, and BSO groups, the Y-Maze test highlighted a significant reduction in SPA for rats in the MLG group one-month post-stereotaxic surgery (*p* < 0.05). Nonetheless, the ANOVA analysis of SPA revealed no significant difference between these groups and the CTR group, irrespective of the recovery period (Figure 2A).

#### 3.2.2. Effects on the Recognition Memory in the NOR Test

After one week or one-month of stereotaxic surgery, the object recognition test demonstrated a significant decrease in the recognition index for the MLG and BA groups compared to the CTR group (*p* < 0.01). Furthermore, after one month of the recovery period, the recognition index remained significantly lower in the MLG group in comparison to the LPS and BSO groups (*p* = 0.0055 and *p* = 0.0006, respectively). During this period, the animals in the BA group exhibited a reduced recognition index compared to the BSO group (*p* = 0.0159). However, the analysis of this parameter showed no significant difference between these groups and the CTR group, irrespective of the recovery period (Figure 2B).

#### 3.2.3. Effects on Spatial Performance Memory in the MWM Test

Throughout the 5 days of training sessions in the MWM test, there were no discernible differences in the latency to reach the submerged platform among the CTR, LPS, OKA, and BA groups. This lack of distinction persisted regardless of the recovery period. However, an evident learning delay emerged in the MLG group during the 5th training session, one week after stereotaxic surgery (*p* = 0.021 compared to the Control group) (Figure 2C). This delay was further exacerbated one month after treatment (Figure 2D), with MLG-treated animals exhibiting significantly higher latency times to reach the submerged platform during the 4th and 5th learning sessions (*p* = 0.0327 and *p* = 0.0056, respectively).

During a probe trial, one hour after the 5th training session (Figure 2E), all groups displayed memory of the platform location, as indicated by their preference for the target quadrant, evidenced by both distance and time spent in that quadrant. Importantly, average swimming speed did not exhibit significant differences between treatment groups, indicating similar motivation and water navigation abilities across all groups.

#### 3.2.4. Cognition Z-Score

To confirm the long-term effect of the treatments on the cognitive deficits, we calculated a cognition Z-score based on the three tests utilized and compared the rats’ performance one week and one-month post-stereotaxic surgery. As depicted in Figure 2F, while the LPS, OKA, and BSO groups exhibited enhanced cognitive performance following one month of stereotaxic surgery, the BA and MLG groups showed either relative stability or a decline in this parameter. Compared to control animals, the disparity in Z-scores with these two groups significantly widened during this timeframe (1W: CTR vs. BA, *p* = 0.0200; CTR vs. MLG, *p* = 0.0079; 1M: CTR vs. BA, *p* = 0.0048; CTR vs. MLG, *p* = 0.0048). These findings suggest progressive and irreversible cognitive deficits in the MLG and BA groups, induced AD-like in rat models.

### 3.3. Biochemical Analysis

Table 1 lists the mean ± SEM of MDA, NO levels, CAT, and SOD activities in the PFC and HIP of treated groups, either two weeks or six weeks’ post-stereotaxic surgery. Subsequent analyses will exclusively focus on comparisons relative to the control group.

#### 3.3.1. MDA Levels

The result of two-way ANOVA (Treatment x Time) of the MDA levels demonstrated a significant effect of the treatment in the PFC (F(5.60) = 13.68; *p* < 0.001) and the HIP (F(5.60) = 27.99; *p*< 0.001) brain subregions. However, no significant effects of time or interaction between the two factors were noted within the two brain subregions. Post-hoc analyses revealed that the treatment effect, after a 2-weeks recovery period, was attributed to a significant increase in MDA levels among the BSO and MLG groups in the PFC (*p* = 0.008 and *p* < 0.001, respectively), and among the BSO, MLG, and BA groups in the HIP (*p* < 0.001, *p* = 0.021 and *p* < 0.001, respectively), compared to the control group. This elevation persisted solely in the MLG group in the PFC (*p* < 0.001), and in the BA and MLG groups in the HIP (*p* = 0.005 and *p* < 0.001, respectively), after 6 weeks’ post-stereotaxic surgery. However, no differences in this parameter were observed between the LPS, OKA groups, and the CTR group in both the PFC and the HIP.

#### 3.3.2. NO Levels

In the two tested brain subregions, the two-way ANOVA analysis of the NO levels showed a significant difference among different groups (PFC: F(5.60) = 34.07; *p*< 0.001); HIP (F(5.60) = 15.38; *p*< 0.001)). A time effect (F(1.60) = 52.96; *p*< 0.001; HIP: F(1.60) = 46.48; *p* < 0.001) as well as a treatment x time interaction (PFC: F(5.60) = 6.474; *p* < 0.001; HIP: F(5.60) = 5.238; *p* < 0.001) were also detected. Inter-group analysis after a two weeks of stereotaxic surgery, indicated a significant increase of NO levels in OKA, LPS, BSO, BA and MLG received rats compared to C in the PFC (*p* < 0.001 for all). However, this same statistical test did not show a significant difference in this parameter except between the MLG and CTR groups, six weeks later in the PFC (*p* < 0.001). Relatively similar results were observed in the HIP over time. After two weeks, the NO level significantly increased in groups OKA (*p* < 0.001), LPS (*p* < 0.001), BSO (*p* = 0.005), BA (*p* = 0.002), and MLG (*p* < 0.001) compared to the control. However, this difference was only observed for the MLG group (*p* = 0.002 vs. CTR) after six weeks of stereotaxic surgery. Regarding the time effect, following a 6-week recovery period, a significant reduction in NO levels was observed in the OKA (*p* = 0.005), LPS (*p* < 0.001), BA (*p* = 0.037), and MLG (*p* = 0.002) groups within the PFC. In the HIP, however, this reduction was evident only in the OKA (*p* = 0.002) and LPS (*p* < 0.001) groups.

#### 3.3.3. CAT Activity

Regarding the CAT activity, two-way ANOVA analysis also demonstrated a significant difference among groups in the PFC (F (5.60) = 8.896; *p* < 0.001) and the HIP (F (5.60) = 14.91; *p* < 0.001) brain subregions. In these two structures, a time effect was also detected (PFC: F(1.60) = 18.39; *p* < 0.001; HIP: F(1.60) = 8.550; *p* = 0.049), with no interaction between the factors. After two weeks of stereotaxic surgery, the treatment effect resulted in a significant decrease in the activity of this enzyme in the BSO and MLG groups in the PFC (*p* = 0.042 and *p* = 0.017, respectively), and in the BA and MLG groups in the HIP (*p* = 0.017 and *p* = 0.003, respectively), compared to the control group. These decreases worsened over time in the MLG and BA groups in both structures studied (*p* < 0.001 vs. control). However, no difference was observed between the BSO and CTR groups after six weeks of stereotaxic surgery (*p* = 0.5203). Concurrently, while CAT activity improved over time in all experimental groups, it notably decreased in the MLG group across both structures examined.

#### 3.3.4. SOD Activity

In both of the PFC and HIP, two-way ANOVA analysis showed a significant difference in SOD activity among different groups (F(5.60) = 11.12; *p* < 0.001 and F(5.60) = 16.32; *p* < 0.001, respectively). The results of Tukey post-hoc analysis, after two weeks of stereotaxic surgery demonstrated that BA and MLG treatments significantly decreases the SOD activity in these brain sub-regions compared to the control group (PFC: *p* = 0.044 and *p* =0.003; HIP: *p* = 0.032 and *p* = 0.002, respectively). After six weeks of stereotaxic surgery, this parameter was further reduced in the BA and MLG groups, both in the PFC and HIP (*p* < 0.001 vs. Control). Similarly, in the HIP, SOD activity was significantly decreased in all experimental groups compared to the control (OKA: *p* = 0.043; LPS: *p* = 0.001; BSO: *p* < 0.001; BA: *p* < 0.001; MLG: *p* < 0.001).

Overall, the results demonstrate that the oxidative status in the tested brain subregions, particularly in the BA and MLG groups, was significantly altered. Furthermore, this modification is irreversible and, on the contrary, worsened over time. These observations suggest that oxidative stress is one of the mechanisms involved in the cognitive pathogenicity previously diagnosed by behavioral tests in treated animals.

### 3.4. Histological Analysis

**Figure 3:**
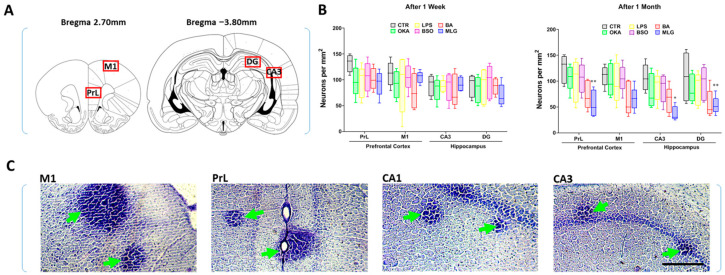
Histologic quantification of neurons in rats’ brain. (**A**) Regions of interest and their anatomical positions: prelimbic cortex (PrL), medial coretx (M1), hippocampal pyramidal cell (CA3) and dental gyrus (DG) (2.70 and −3.80 mm relative to bregma; left hemisphere). (**B**) Number of neurons per mm2 assessed by cell counting in PrL, M1, CA3 and DG subregions. (**C**) Representative Nissl-stained forebrain sections showing potential inflammatory infiltration in the MLG group (green arrows) across the M1, PrL, CA1 and CA3 brain subregions. The data are illustrated as box-and-whisker plots for CTR, OKA, LPS, BSO, BA and MLG groups. Each value represents the mean ± SEM. from 5 animals per group. Symbols Signification: * *p* < 0.05, ** *p* < 0.01 vs. CTR according to Kruskal–Wallis ANOVA, and Dunn’s multiple comparison test. Scale bars: 200 µm. Drawings are adapted from Paxinos & Watson, (2013). All coordinates for the studied subregions were obtained from the rat brain atlas [23].

**Figure 4:**
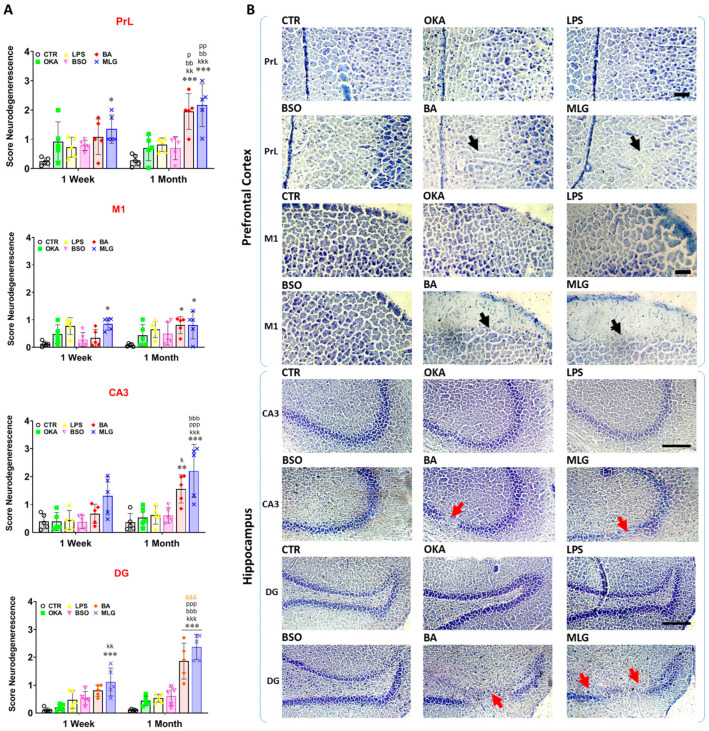
Histologic semi-quantitative analysis of neurodegeneration in rats’ brain. (**A**) Score of Neurodegeneration in prelimbic cortex (PrL), medial coretx (M1), hippocampal pyramidal cell (CA3) and dental gyrus (DG) subregions. (**B**) Representative images of Nissl-stained forebrain sections (2.70 and −3.80 mm relative to bregma; left hemisphere; PrL, M1, CA3, and DG) for CTR, OKA, LPS, BSO, BA, and MLG groups. Cellular loss and areas of neurodegeneration are indicated by black and red arrows, respectively. Each value represents the mean ± SEM. from 5 animals per group. Symbols Signification: * *p* < 0.05, ** *p* < 0.01, *** *p* < 0.001 vs. CTR; ^k^ *p* < 0.05, ^kk^ *p* < 0.01, ^kkk^ *p* < 0.001 vs. AOK; ^p^ *p* < 0.05, ^pp^ *p* < 0.01, ^ppp^ *p* < 0.001 vs. LPS; ^bb^ *p* < 0.01, ^bbb^ *p* < 0.001 vs. BTN; ^δδδ^ *p* < 0.001: 1W vs. 1M (time effect) according to Kruskal–Wallis ANOVA, and Dunn’s multiple comparison test. Scale bars: 50 µm or 150 µm. Drawings are adapted from the rat brain atlas [23].

As shown in Figure 3 and Figure 4, neurodegeneration in brain subregions of all groups was observed when sections were visually analyzed by a scoring system (Figure 4A) or when neurons were counted in respective sections (Figure 3B). Two weeks after stereotaxic surgery, Kruskal-Wallis ANOVA analysis of neurodegeneration, followed by Dunn’s post hoc test, revealed significant neuronal loss in all the tested brain subregions in the MLG group compared to control (Score, DG: *p* < 0.001; PrL: *p* = 0.0198; M1: *p* = 0.0274; Figure 4A). This neurodegeneration was notably more pronounced after six weeks of stereotaxia in the same group of animals (Score, CA3: *p* < 0.001; DG: *p* < 0.001; PrL: *p* < 0.001; M1: *p* = 0.0197; Figure 4A) and was also evident in the BA group (Score, CA3: *p* = 0.0069; DG: *p* < 0.001; PrL: *p* < 0.001; M1: *p* = 0.0297; Figure 4A). Moreover, the number of neurons per mm^2^ was significantly reduced in CA3, DG and PrL hippocampal and cortex limbic subregions–respectively-in MLG group (CA3: *p* = 0.0135; PrL: *p* = 0.0058; Figure 3B). Yet, in all of these brain subregions, no difference was found between groups in the number of neurons after two weeks of the stereotaxic surgery.

Apart from the brain lesions identified in the tested regions, inflammatory foci were also observed in the stained sections (Figure 3C), resulting from immune cell infiltrations into the brain parenchyma, particularly in animals treated with the molecule mixture used in this experiment. These observations suggest that a neuro-inflammatory component was also involved in the cognitive pathogenicity characteristic of AD, providing further evidence of the efficacy of the molecule mixture used in our experiment to model this disease in Wistar rats. Together, these findings consistent with oxidative status changes in the brain in both the BA and MLG groups, indicating a progressive and irreversible toxicity induced by the molecule mixture, as well as by BA injected into the cerebroventricular region of rats.

## 4. Discussion

The fact that AD is still an incurable pathology necessitated and continues to require the development of new animal models, which has greatly contributed to the diversity and abundance of AD models proposed in the literature. But that does not add a point to the effort needed to complete our understanding of the complex and overlapping mechanisms of this pathology. In this context, this work aims to do a comparative study between different pharmacological animal models based on the most reported theories to explain the pathology: Aβ plaque aggregation, tau protein hyperphosphorylation, oxidative stress, and neuroinflammation. We tested four molecules known in the literature to induce each of these disorders in the brain: Oligomeric Aβ1-42, OKA, LPS and BSO, respectively. Additionally, for the first time, we examined the synergistic effect of these four molecules when injected in combination, assessing their impact on the induction of AD-like and cognitive deficits both in the short and long term.

The accumulation of Aβ in the brain is one of the persistent markers of AD pathogenesis that ultimately leads to inflammation, oxidative stress, and progressive and irreversible cognitive dysfunction. Therefore, the icv injection of toxic soluble species of Aβ1-42 (oligomers, protofibrils, and fibrils) into the rat brain has been widely used as a model to study the persistent induced events of AD. In line with several studies [24,33,34], we found that the icv injection of oligomeric Aβ1-42 induced –after one week- a significant decrease in spatial memory, recognition memory, and working memory in the MWM, ORT, and Y-Maze tests, respectively. These same deficits were also observed in the OKA, the BSO and the LPS-treated animals, but the decrease compared to control was not significant. However, other studies using higher doses or intrahippocampal injection of these same molecules have demonstrated more significant effects on the cognitive functions of animals [35]. Borbely et al. demonstrated that intrahippocampal administration of synthetic Aβ peptides simultaneously decreases both the spatial learning capacity in MWM and the density of the dendritic column and synaptic plasticity (LTP) in the CA1 region of the rat HIP [36]. Studies of the distribution of fluorescent-labeled Aβ1-42 in the brain showed that soluble Aβ-species diffused into all parts of the rat brain [24]. Our results of histological studies both short-term and long-term (irreversible decreased number of viable neurons, increased neurodegeneration score, especially in hippocampal substructures: DG and CA3) confirm the behavioral observations and also supported that icv administration of well-characterized soluble toxic Aβ molecules into the rat brain provides a reliable rat AD-model.

The comparison of the toxic effects of the four molecules used in this study oligomeric Aβ1-42, OKA, LPS, and BSO also revealed that the cognitive deficits and neurodegeneration observed in animals receiving Aβ1-42 persist even one month after injection. In contrast, these effects were relatively diminished or even improved one-month post-stereotaxic surgery in rats that received the other molecules. Other studies have shown that Aβ accumulation in the brain is associated with long-lasting inflammation, oxidative stress, and mitochondrial dysfunction, which are positively correlated with the characteristic brain neurodegeneration of AD [36,37,38]. In our study, all molecules induced short-term oxidative stress in the brain, as indicated by increased levels of NO and MDA and decreased activity of CAT and SOD. Consistent with several studies, this effect was highly significant for BSO and Aβ1-42 [33,39]. Interestingly, one month after injection, oxidative stress persisted only in animals that received Aβ1-42, particularly in the HIP, whereas this effect was observed only as a reduction in SOD activity in animals that received BSO. These findings further confirm the efficacy of intraventricular Aβ1-42 administration as a reliable animal model of AD.

Another original point of this study is the co-injection of four molecules as a mixture. Notably, animals receiving this mixture exhibited the most pronounced cognitive deficits compared to other groups, showing significant impairments in working memory, object recognition memory, and spatial memory. These cognitive deficits were also linked to substantial oxidative stress and neurodegeneration in the examined brain structures. Interestingly, this group of animals not only experienced persistent impairments but also demonstrated a progressive decline over time, with all measured parameters worsening one month after stereotaxic surgery. To our knowledge, this mixture has not been used previously to model AD in rats. In contrast, earlier studies have used different combinations to show that a single molecule is inadequate to induce AD-like symptoms. For instance, Lecanu et al. demonstrated that infusing Fe^2+^, Aβ42, and buthionine-sulfoximine (FAB), but not Aβ42 alone or in combination with Fe^2+^, into the left cerebral ventricle of Long-Evans rats for 4 weeks led to memory impairment and increased levels of hyperphosphorylated Tau protein in CSF [16]. Animals infused with FAB exhibited thioflavin-S-positive amyloid deposits, hyperphosphorylated Tau protein, neuronal loss, and gliosis. Conversely, animals treated with Aβ42, Fe^2+^, or buthionine-sulfoximine alone or in various combinations did not display the same histopathological changes seen with FAB. This evidence suggests that Aβ42 alone is insufficient to induce AD-like symptoms and supports a model where a compromised antioxidant defense system in the brain is necessary for Aβ42 to trigger oxidative stress, resulting in the observed histopathological changes and memory deficits [16]. Therefore, the mixture used in our study is considered one of the best choices for modeling the symptomatology of AD and obtaining a comprehensive clinical picture of the pathology. This makes it an ideal animal model for therapeutic research and for studying the complex pathophysiology of the disease. However, it is also important to acknowledge some major limitations of our approach. Specifically, we did not measure tau protein phosphorylation levels, Aβ levels, or assess certain markers of neuroinflammation. Future studies will be necessary to validate our results and complete this assessment.

**Figure 5:**
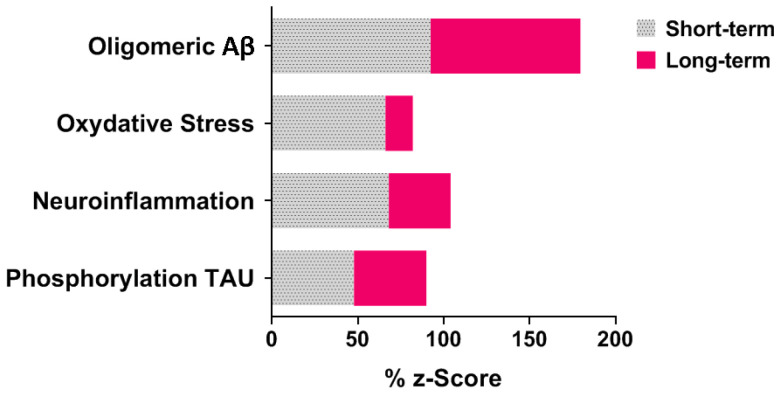
Hypothetical percentages of involvement of pathophysiology triggering AD. The Z-scores, expressed as percentages, were calculated for each experimental group after one week (short-term) or one month (long-term) post-stereotaxic surgery. These Z-scores serve as indicators for the corresponding pathophysiological hypotheses related to AD. For instance, the Z-score associated with the beta-amyloid accumulation hypothesis reflects the average of the Z-scores across all behavioral parameters (Cognition Z-score), biochemical parameters (Oxidative Stress Z-score), and histological parameters (Neurodegeneration Z-score).

In light of the key pathophysiological mechanisms underlying AD and to understand the synergistic effects of the inducing agents, we propose new therapeutic strategies that target each mechanism with specific pharmacological interventions. We have developed a hypothetical model illustrating the percentage involvement of each pathophysiological mechanism in triggering AD (Figure 5). This model is based on a Z-score calculated using the algebraic sum of the parameters measured for each experimental group (Behavioral Score, Biochemical Score, and Histological Score). These scores have been converted into percentages for parameters measured in the short term or long term (one week or one-month post-stereotaxic surgery, respectively). This approach aims to provide comprehensive therapeutic strategies addressing the multifactorial nature of AD and to tailor interventions according to the stage of disease progression. We hypothesized that the therapeutic management of AD should be tailored according to the severity of symptoms. In cases of early diagnosis, it is recommended that therapeutic intervention initially focus on reducing Aβ accumulation in the brain, followed by the inhibition of neuroinflammation and oxidative stress. At an early stage of the disease, targeting tau protein phosphorylation is suggested to be of lower priority. Conversely, in advanced stages of the disease, treatments should concentrate on reducing tau hyperphosphorylation. At this stage, the involvement of neuroinflammation and oxidative stress is generally less pronounced. Nevertheless, it is crucial to maintain consistent attention to anti-Aβ treatments throughout all stages of the disease. All these remarks and suggestions remain to be confirmed by more robust and in-depth studies.

## 5. Conclusions

Data from this study demonstrated that the icv administration of the Aβ1-42, LPS, OKA, and BSO mixture in rats induced persistent and progressive deficits in working memory, recognition memory, and spatial memory. These behavioral impairments in memory and learning were accompanied by irreversible and significant changes in oxidative stress and neurodegeneration, particularly in the HIP brain structure. Additionally, our results improve the ‘icv-administered Aβ’ rat model using well-characterized Aβ1-42 oligomers. Moreover, considering the rationale used to develop the mixture model, the data presented herein support a unifying ‘Amyloid-Oxidative-Tau Phosphorylation-Inflammation’ hypothesis to explain the onset and progression of AD. This study also provides suggestions for therapeutic strategies for the effective management of AD, which should take into account the severity of the disease.

## Figures and Tables

**Table 1:** Oxidative status of the brain in rats after 2 or 6 weeks of stereotaxic surgery.

	Brain Subregions	Markers of OS	CTR	OKA	LPS	BSO	BA	MLG
**After 2 Weeks**	**PFC**	*MDA*	1.08 ± 0.2	1.37 ± 0.2	1.99 ± 0.2	3.51 ± 0.5 **	3.08 ± 0.5	4.40 ± 0.5 ***
		*NO*	39.92 ± 7.6	154.32 ± 10.8 ***	169.69 ± 9.4 ***	117.06 ± 14.7 ***	156.32 ± 12.2 ***	210.46 ± 10.6 ***
		*CAT*	5.22 ± 0.5	4.04 ± 0.5	3.75 ± 0.4	2.97 ± 0.7	3.00 ± 0.7	2.17 ± 0.4
		*SOD*	8.06 ± 0.7	6.67 ± 1.4	5.77 ± 1.1	4.09 ± 1.0	3.92 ± 0.9	2.68 ± 0.3
	**HIP**	*MDA*	2.02 ± 0.1	2.46 ± 0.2	3.04 ± 0.4	6.02 ± 0.3 ***	5.03 ± 0.4 *	7.20 ± 0.6 ***
		*NO*	79.39 ± 8.0	168.85 ± 11.8 ***	169.21 ± 8.8 ***	143.18 ± 9.5 **	146.32 ± 13.9 **	195.82 ± 8.9 ***
		*CAT*	4.87 ± 0.5	4.17 ± 0.8	3.24 ± 0.5	3.14 ± 0.5	1.94 ± 0.4	1.42 ± 0.2 *
		*SOD*	10.04 ± 1.1	7.73 ± 1.0	7.62 ± 0.9	6.79 ± 1.3	4.74 ± 0.5	3.17 ± 0.7
**After 6 Weeks**	**PFC**	*MDA*	1.71 ± 0.2	1.76 ± 0.4	1.65 ± 0.2	145.45 ± 9.8	2.918 ± 0.5	4.28 ± 0.6**
		*NO*	64.81 ± 9.1	96.97 ± 4.5 ^δδ^	85.76 ± 12.2 ^δδδ^	69.59 ± 9.1	105.15 ± 9.7 ^δ^	145.45 ± 9.8 *** ^δδ^
		*CAT*	6.62 ± 0.6	6.10 ± 0.7	6.10 ± 0.7	5.05 ± 0.8	4.42 ± 0.5	1.95 ± 0.4 ***
		*SOD*	9.46 ± 0.9	7.42 ± 1.2	6.42 ± 1.3	5.55 ± 0.9	3.52 ± 0.4	2.10 ± 0.4
	**HIP**	*MDA*	2.33 ± 0.4	3.09 ± 0.5	2.88 ± 0.4	4.75 ± 0.8	5.70 ± 1.0 **	8.36 ± 0.6 ***
		*NO*	86.47 ± 7.2	100.39 ± 16.4 ^δδ^	79.51 ± 8.4 ^δδδ^	94.18 ± 7.9	131.32 ± 9.4	154.78 ± 14.7 **
		*CAT*	6.90 ± 0.7	5.78 ± 0.5	4.34 ± 0.8	4.47 ± 0.8	2.21 ± 0.4	1.36 ± 0.2 ***
		*SOD*	16.04 ± 1.9	10.92 ± 1.1	8.83 ± 1.5	8.46 ± 1.7	5.58 ± 0.8	2.99 ± 0.5

Oxidative stress (OS) was assessed after 2 or 6 weeks of stereotaxic surgery (recovery + behaviors test periods) in the hippocampus (HIP) and the prefrontal cortex (PFC) of rats. The results are expressed as mean ± SEM (from at least 5–6 animals per group) of the MDA: Malondialdehyde (nmol/g of homogenate); NO: Nitrite content (μmol/g of homogenate); CAT: Catalase activity (unit/min/mg of protein); SOD: Superoxyde Dismutase activity (unit per mg of protein). * vs. CTR and (^δ^) for the time effect. One symbol: *p* < 0.05; Two symbols: *p* < 0.01; Three symbols: *p* < 0.001 according to Two –ways ANOVA (Treatment × Time) followed by Tukey’s test.

## Data Availability

The original contributions presented in the study are included in the article.
